# Differences in location of cerebral white matter hyperintensities in children and adults living with a treated HIV infection: A retrospective cohort comparison

**DOI:** 10.1371/journal.pone.0241438

**Published:** 2020-10-28

**Authors:** Jason G. van Genderen, Malon Van den Hof, Anders C. Boyd, Matthan W. A. Caan, Ferdinand W. N. M. Wit, Peter Reiss, Dasja Pajkrt

**Affiliations:** 1 Department of Pediatric Infectious Diseases, Emma Children’s Hospital, Amsterdam UMC, University of Amsterdam, Amsterdam, The Netherlands; 2 HIV Monitoring Foundation, Amsterdam, The Netherlands; 3 Public Health Service of Amsterdam, Department of Infectious Diseases, Amsterdam, The Netherlands; 4 Department of Biomedical Engineering and Physics, Amsterdam UMC, University of Amsterdam, Amsterdam, The Netherlands; 5 Department of Radiology, Amsterdam UMC, University of Amsterdam, Amsterdam, The Netherlands; 6 Amsterdam Institute for Global Health and Development (AIGHD), Amsterdam, The Netherlands; 7 Department of Global Health, Amsterdam UMC, University of Amsterdam, Amsterdam, The Netherlands; University at Buffalo, UNITED STATES

## Abstract

Cerebral white matter hyperintensities (WMH) persist in children and adults living with HIV, despite effective combination antiretroviral therapy (cART). As age and principal routes of transmission differ between children (perinatally) and adults (behaviorally), comparing the characteristics and determinants of WMH between these populations may increase our understanding of the pathophysiology of WMH. From separate cohorts of 31 children (NOVICE) and 74 adults (AGE_h_IV), we cross-sectionally assessed total WMH volume and number of WMH per location (periventricular vs. deep) using fluid-attenuated inversion recovery (FLAIR) MRI images. WMH were either periventricular when within 10mm of the lateral ventricles, or deep otherwise. We assessed patient- or HIV-related determinants of total WMH volume (adjusted for intracranial volume) and location of WMH using logistic regression, while stratifying on children and adults. At enrollment, median age of participants was 13.8 years (IQR 11.4–15.9) for children and 53.4 years (IQR 48.3–60.8) for adults and 27/31 children (87%) and 74/74 adults (100%) had an HIV RNA viral load <200 copies/mL. WMH were present in 16/27 (52%) children and 74/74 adults (100%). The prevalence of deep WMH was not different between groups, (16/16 [100%] in children vs. 71/74 [96%] in adults, p = 0,999), yet periventricular WMH were more prevalent in adults (74/74 [100%]) compared to children (9/16; 56%) (*p*<0.001). Median WMH volume was higher in adults compared to children (1182 mm^3^ [425–2617] vs. 109 mm^3^ [61.7–625], *p*<0.001). In children, boys were more likely to have deep WMH compared to girls. In adults, older age was associated with higher total WMH volume, and age, hypertension and lower CD4^+^ T-lymphocyte nadir with a higher number of periventricular WMH. Our findings suggest that the location of WMH differs between children and adults living with HIV, hinting at a different underlying pathogenesis.

## Introduction

The widespread use of combination antiretroviral therapy (cART) has resulted in a substantial decline in the incidence of severe HIV-related neurological complications, including HIV-encephalopathy in children with perinatally acquired HIV infection (PHIV) and HIV-associated dementia in adults with behaviorally acquired HIV infection [1, 2].

Cerebral white matter hyperintensities (WMH) have been reported to be more prevalent in children and adults living with HIV on effective cART compared to their respective healthy controls [3–5]. In adults, WMH are associated with poorer cognitive performance, namely attention and learning as compared to adults without HIV [6, 7], which may lead to poorer cART adherence and increased need of social services [8, 9].

The pathogenesis of HIV-associated WMH is not fully understood. Potential pathophysiological mechanisms include direct vascular pathology, ongoing effects from HIV-associated neuroinflammation, lasting effects of neuronal damage induced during untreated infection, and neurotoxic effects of cART [10–12].

Magnetic Resonance Imaging (MRI) scans are sensitive in detecting subtle HIV-related brain changes; fluid-attenuated inversion recovery (FLAIR) is commonly used to detect WMH [13, 14]. Studies of brain structure and function, including those addressing WMH, are limited in children on cART [13]. One study from our research group found a higher WMH volume in PHIV children compared to healthy matched controls [3], whereas a recent case-control study performed in Zambia found no difference in WMH prevalence [15]. Another study, without a controlled comparison, reported a 50% prevalence of WMH in a group of children who were investigated because of suspected HIV-related brain disease [16].

In contrast to children, more studies have investigated brain structure, including WMH, in adults. In our own study, including middle-aged adults with suppressed viremia on cART, we observed a higher total WMH volume compared to controls, which was associated with poorer global cognitive function [5]. In another study, we did not find a significant difference between adults living with HIV and controls in the change of WMH volume during two years of follow-up [17]. One study reported HIV-status to be an independent risk factor for cerebral small vessel disease, including WMH [4]. Finally, two smaller studies, however, did not report HIV-status to be associated with prevalence and volume of WMH [18, 19].

Thus far, no studies have attempted to compare prevalence, number, volume and distribution of WMH between children and adults living with HIV. To increase pathophysiological understanding of cerebral WMH in people living with HIV, we explored their possible relationship with age and mode of HIV acquisition, by comparing the presence, size and location of WMH between our ongoing cohort studies of children and adults living with HIV. We hypothesized that WMH would be more prevalent and differently distributed in adults compared to children, and that adults would have a greater degree of periventricular WMH in view of the known relationship with age [20].

## Materials and methods

### Study design and participants

We used anonymized data from participants at cohort entry from two cohort studies: the Neurological, cOgnitive and VIsual performance in perinatally HIV-infected ChildrEn (NOVICE) study and the neuroimaging substudy of the AGE_h_IV cohort study [3, 5]. The NOVICE study investigates neurological, cognitive and visual performance in PHIV-infected children compared to controls matched for age, sex and socio-economic status [21]. The AGE_h_IV cohort study is an ongoing prospective cohort study evaluating the occurrence of age-related comorbidities in HIV-1-positive and demographically and lifestyle-similar HIV-negative adults 45 years of age or older, the details of which have been previously published (22). A neuroimaging substudy evaluated neurocognitive outcomes in a subset of male participants who had sustained HIV-viremia suppression for at least 12 months [5].

The following exclusion criteria had been used for both cohorts: current or past neurological or psychiatric disorders not associated with HIV, a history of traumatic brain injury resulting a loss of consciousness of more than 30 minutes, intracerebral neoplasms and MRI contraindications including metal implants or claustrophobia; the complete criteria for non-inclusion have been previously published in detail [3, 5]. The ethics committee of the Amsterdam University Medical Centers approved both study protocols. Both cohorts obtained written informed consent from all participants older than 12 years and from parents of participants younger than 18 years of age. The AGE_h_IV cohort study is registered at ClinicalTrials.gov (identifier NCT01466582) and the NOVICE cohort study at the trial Dutch Trial registry (identifier NTR4074).

### Demographic and HIV-related variables

Data on historical HIV- and-cART related parameters were provided by the Dutch HIV Monitoring Foundation [21, 22]. Additionally, adult participants completed an extensive standardized questionnaire concerning a wide range of demographics, medical characteristics and lifestyle-related factors [23].

### MRI data acquisition

At enrollment one MRI scan with different modalities was performed in all pediatric and adult participants initially with a 3.0 Tesla Intera system and subsequently with a 3.0 Tesla Ingenia system (Philips Healthcare, Best, the Netherlands) due to a scanner software upgrade. Three-dimensional fluid-attenuated inversion recovery (FLAIR) scans were performed to image both periventricular and deep WMH, using the following MRI scanning parameters: echo time = 356 ms in children and 355 ms in adults; repetition time = 4,800 ms; inversion time = 1,650 ms; field of view = 240 × 240 mm^2^ in children and 250 × 250 mm^2^ in adults; 321 sagittal slices of 0.56 mm thickness; 1.0 × 1.0 mm^2^ in-plane resolution in children and 1.1 × 1.1 mm^2^ in adults (3,5).

### MRI image processing

**Fig 1:**
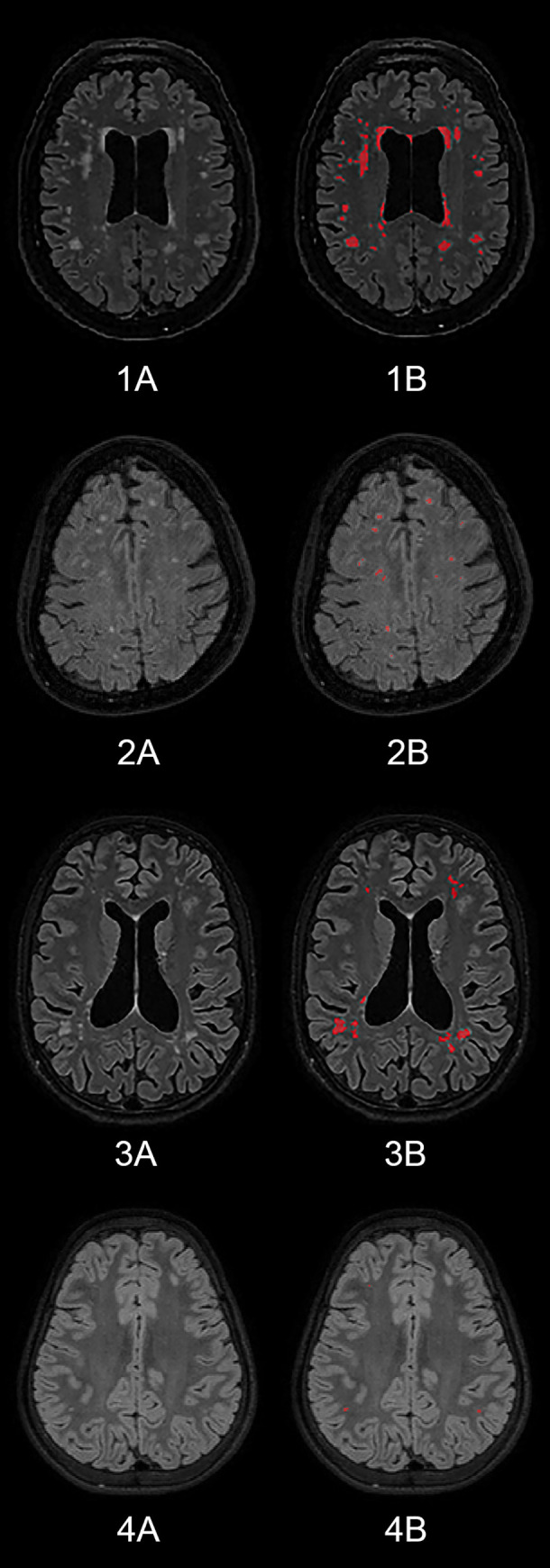
Image segmentations. The images show axial MRI planes of two adults (1 and 2) and two children (3 and 4). In the images coded “B” WMH have been manually adjusted and labeled (red color).

We anonymized all MRI data prior to analysis and evaluated them for eligibility, while excluding those in which excessive head motion occured. A neuroradiologist reviewed all MRI scans for incidentalomas [3, 24]. For the purpose of the current analysis, one investigator (JvG) reviewed all FLAIR image segmentations and manually adjusted these segmentations (by labeling and delabeling WMH [Fig 1]) for false negative or false positive lesions using the segmentation software ITK-SNAP (version 3.2, Philadelphia, PA; Salt Lake City, UT, USA) [25].

We assessed the following outcome variables: total volume of WMH, total number of WMH and number of WMH per location (periventricular vs. deep). We assessed the number of WMH by manually counting all individual lesions within an MRI FLAIR. We obtained the total segmented WMH volume through ITK-SNAP. We assessed the location of WMH by defining lesions to be either periventricular or deep WMH. We defined lesions as periventricular lesions when they were located within 10 mm of the lateral ventricles [26]. All other WMH were defined as deep.

### Statistical analysis

We performed statistical analyses using R version 3.5.1 (R Core team, Vienna, Austria) [27]. We considered *p* < 0.05 as statistically significant.

We compared demographic variables between children and adults using the Mann-Whitney *U* test for continuous data and Fisher’s exact test for categorical data.

We adjusted WMH volume for intracranial volumes (ICV), i.e. WMH to ICV ratio, for direct comparison between groups. The ICV had been previously calculated automatically [3, 5]. Due to non-normal distributions, we dichotomized WMH volume and the number of WMH per location based on the median level. We used univariable logistic regression to determine associations of high volume and high number per location of WMH, with the following demographic, HIV- and cART-related characteristics: age, gender, hypertension in adults and measured high blood pressure in children, known duration of HIV infection, CD4^+^ T-lymphocyte nadir, and duration of antiretroviral treatment. We defined high blood pressure in children based on measured blood pressure according to American Association of Pediatrics guidelines [28]. Hypertension in adults was defined as a diastolic blood pressure ≥ 90 mmHg and/or systolic blood pressure ≥ 140 mmHg, or use of antihypertensive medication. We performed additional analyses in children to asses the associations between location of WMH and adoption status, total Intelligence Quotient (IQ) and severity of HIV history defined by Center of Disease Control and Prevention (CDC) classification.

Given the low numbers of participants included in analyses, the risk of obtaining unrealistically high odds ratios (OR) and inflated type I error, i.e. “sparse data bias” [29], is increased. In order to mitigate this bias, we applied penalized regression through the use of data augmentation techniques [29, 30]. Briefly, we constructed non-informative prior distributions of the OR based on an F(2,2) distribution (i.e. OR = 1; 95%CI = 1/39–39), which were appended to the actual study data to estimate a *posterior* OR. This estimate allows ORs to be anchored on realistic estimates in the presence of sparse-data. As the amount of data increases, the influence of the prior distribution becomes weaker. A *posterior* OR and its associated 95% confidence interval were calculated using the “R PLRDA” and “plogit” programs in R [29]. Multivariable analysis was precluded by the low numbers of participants.

## Results

### Participant characteristics

From a total of 35 children and 74 adults included, we excluded four scans from NOVICE participants because of excessive head motion leading to poor quality. Thus, we analyzed data from 31 children and 74 adults. Table 1 shows the characteristics of participants at time of enrollment into their respective cohort. Due to missing data on ICV, we excluded one child and two adults from the analysis of adjusted total WMH volume for which the WMH to ICV ratio was required.

**Table 1:** Demographic, HIV- and treatment-related participant characteristics.

Characteristics	Children (n = 31)	Adults (n = 74)	*p* value
Age^¤^	13.8 (11.4–15.9)	53.4 (48.3–60.8)	**< 0.001**^a^
Male gender	16 (52)	74 (100)	**< 0.001**^b^
**Country of Birth𝄐**			**< 0.001**^b^
Netherlands	11 (36)	61 (85)	
Sub-Saharan Africa	15 (48)	2 (3)	
Suriname	2 (6)	3 (4)	
Other	3 (10)	6 (8)	
**HIV- and treatment-related characteristics**			
Age at HIV diagnosis^¤^	1.2 (0.6–4.9)	42.6 (37.2–46.2)	**< 0.001**^a^
Age at treatment initiation^¤^	2.2 (0.9–5.2)	45.0 (38.5–50.2)	**< 0.001**^a^
Currently on cART	28 (90)	74 (100)	**0.024**^b^
Duration of ART	11.8 (7.7–14.5)	10.9 (4.3–14.9)	0.564^a^
CD4^+^ nadir^#^	430 (250–750)	160 (57.5–232.5)	–
CD4^+^ nadir %	18 (13–28)		
CD4^+^ nadir *Z* score^ω^	–0.7 (–1.5 to 0.4)		
HIV VL zenith/prior to treatment	5.5 (5.1–5.9)	5.0 (4.4–5.6)	
**Cardiovascular risk**			
High blood pressure~/hypertension	5 (16)	28 (38)	0.008
**HIV viral load at MRI**			0.007^b^
Detectable	4 (13)	0 (0)	
Undetectable*	27 (87)	74 (100)	
**MRI characteristics**			
Philips Intera / Ingenia	31 (100) / 0 (0)	72 (97) / 2 (3)	0.999^b^
MRI FLAIR good quality allowing proper assessment	27 (87)	74 (100)	–

Abbreviations: ART = antiretroviral therapy (including prior mono or dual therapy), cART = combination antiretroviral therapy; treatment in adults is either cART or ART; FLAIR = fluid-attenuated inversion recovery; HIV = human immunodeficiency virus; MRI = magnetic resonance imaging; VL = viral load zenith in children and prior to treatment in adults (logarithmic value unit: copies/ml); Values noted in amount and percentage n(%) or median and inter-quartile range (IQR)

Statistical tests

^a^ = Mann-Whitney *U* test

^b^ = Fisher's exact test

^¤^ = age in years

**𝄐** = two adults excluded for not filling in their questionnaire; ~ = in children this is measured high blood pressure at enrollment and defined per guidelines of American Association of Pediatrics

^ω^ = age-adjusted *Z* score

^#^ = measured in cells/μL

* = defined as viral load < 200 copies/ml

The median age at enrollment was 13.8 years (IQR 11.4–15.9) in children vs. 53.4 years (IQR 48.3–60.8) in adults. The median age at HIV diagnosis was 1.2 years in children (IQR 0.6–4.9) vs. 42.6 years (IQR 37.2–46.2) in adults. The overall duration of antiretroviral treatment was comparable (*p* = 0.564) with a median of 11.8 years (IQR 7.7–14.5) and 10.9 years (IQR 4.3–14.9) in children and adults, respectively. All 74 adults had an undetectable viral load with < 200 copies/ml. Three (10%) of the children were not on cART at time of enrollment due to personal reasons and four children (13%) had a detectable viral load (range: 5485–188525 copies/ml). In the five children in whom we measured a high blood pressure, the cause of the hypertension was stress related and thus interpreted as incidentally high blood pressure. There was no treatment initiated to lower the blood pressure. Additional participants’ characteristics–used as variables to assess for determinants in children–are provided as S1 Table.

### WMH assessment

Adults had a higher prevalence of WMH compared to children. Adults had more WMH and a higher total WMH volume compared to children. Whereas the prevalence of deep WMH was comparable between adults and children, the median number of deep WMH was higher, albeit non-significantly, in adults. Adults had both a significantly higher prevalence and median number of periventricular WMH than children (Table 2). In the pediatric participants two children had a history of HIV-encephalopathy and one child had an opportunistic Cytomegalovirus-encephalitis. A sensitivity analysis without these three participants did not alter the results in Tables 2 and 3 (S2 and S3 Tables).

**Table 2:** WMH assessment between children and adults.

	Children (n = 27)	Adults (n = 74)	*p* value
**Prevalence of WMH**			
Participants with WMH	16/27 (59)	74/74 (100)	**< 0.001**^a^
**Quantitative and volumetric analyses**			
Absolute number of WMH	5.0 (2.0–12.5)	18 (9.3–37.5)	**< 0.001**^b^
Intracranial volume^#^	1.52 (1.27–1.58)	1.69 (1.59–1.80)	
Total WMH volume *	109 (61.7–652)	1182 (425–2617)	**< 0.001**^b^^#^
**Location of WMH**			
Participants with deep WMH	16/16 (100)	71/74 (96)	0.999^a^
Participants with periventricular WMH	9/16 (56)	74/74 (100)	**< 0.001**^a^
Absolute number deep WMH	3.0 (2.0–11.3)	11 (3.0–27.8)	0.078^b^
Absolute number periventricular WMH	1.0 (0.0–2.3)	7.0 (6.0–10.8)	**< 0.001**^b^

Abbreviations: HIV = human immunodeficiency virus

WMH = white matter hyperintensities

Values noted in amount and percentage n(%) or median and inter-quartile range (IQR)

Statistical tests

^a^ = Fisher's exact test

^b^ = Mann-Whitney U test

^#^ unit = *10^6^ μL

* unit = mm^3^

^#^ adjusted p value (WMH to ICV ratio)

**Table 3:** Univariable logistic regression analyses on ICV-adjusted WMH volume and location in children.

	ICV-adjusted WMH volume in children (higher versus lower than median)	Presence of deep WMH in children (yes versus no)	Presence of periventricular WMH in children (yes versus no)
	OR (95% CI)	OR (95% CI)	OR (95% CI)
Age	0.97 (0.74–1.27)	0.88 (0.67–1.14)	0.97 (0.74–1.27)
Female gender	0.28 (0.06–1.23)	0.17 (0.04–0.74)	0.84 (0.21–3.33)
High blood pressure	1.00 (0.18–5.42)	1.84 (0.31–10.81)	1.82 (0.32–10.26)
Known HIV years	1.24 (0.84–1.83)	1.09 (0.76–1.55)	1.38 (0.94–2.03)
Age at treatment initiation	1.11 (0.89–1.39)	1.02 (0.84–1.02)	1.08 (0.88–1.32)
Treatment years	0.89 (0.74–1.06)	0.94 (0.80–1.11)	0.92 (0.77–1.08)
CD4^+^ nadir *Z* score	0.69 (0.31–1.53)	0.74 (0.35–1.56)	0.46 (0.16–1.32)
HIV VL zenith	0.40 (0.09–1.72)	0.69 (0.19–2.58)	0.80 (0.21–3.07)
CDC NA		1.81 (0.30–10.78)	0.29 (0.03–2.67)
CDC B		0.66 (0.16–2.66)	1.33 (0.31–5.72)
CDC C		1.05 (0.24–4.63)	1.60 (0.34–7.43)
Adopted		0.90 (0.12–6.62)	1.51 (0.20–11.66)
Total IQ		1.04 (0.98–1.11)	1.04 (0.97–1.10)

Univariable logistic regression models with applied penalized regression using data augmentation. ICV-adjusted WMH volume in children was dichotomized by median split. High blood pressure measured at enrollment defined per guidelines of American Association of Pediatrics; CD4^+^ nadir *Z* score is age-adjusted. Abbreviations: CDC = Center for Disease Control and Prevention, A = minimal symptoms to AIDS, B = moderate symptoms C = severe symptoms or AIDS; HIV = human immunodeficiency virus; ICV = intracranial volume; IQ = intelligent quotient; OR = odds ratio; VL = viral load (logarithmic value; unit: copies/ml); WMH = white matter hyperintensities

### Determinants of WMH volume and location

In children, female gender was associated with a lower prevalence of deep WMH. We found no other significant association in children between patient- or HIV-related characteristics and WMH volume or location (Table 3).

In adults, higher age was associated with an ICV-adjusted WMH volume above the median. In univariable analysis, higher age and hypertension were associated with a number of periventricular WMH above the median. A higher CD4+ T-lymphocyte nadir (per 100 cells/μL) was associated with a number of periventricular WMH below the median (Table 4).

**Table 4:** Univariable logistic regression analyses on ICV-adjusted WMH volume and location in adults.

	ICV-adjusted WMH volume in adults (higher versus lower than median)	Number of deep WMH in adults (higher versus lower than median)	Number of periventricular WMH in adults (higher versus lower than median)
	OR (95% CI)	OR (95% CI)	OR (95% CI)
age	1.18 (1.08–1.28)	1.03 (0.96–1.10)	1.10 (1.03–1.18)
hypertension	1.91 (0.76–4.79)	1.75 (0.70–4.39)	2.93 (1.13–7.59)
known HIV years	1.06 (0.99–1.15)	1.02 (0.94–1.09)	1.04 (0.96–1.12)
treatment years	1.12 (1.02–1.22)	1.03 (0.95–1.12)	1.07 (0.98–1.16)
CD4^+^ nadir	0.62 (0.76–4.79)	1.17 (0.82–1.66)	0.53 (0.33–0.85)
HIV VL prior to treatment	0.79 (0.42–1.48)	0.75 (0.40–1.42)	0.84 (0.44–1.61)

Univariable logistic regression models with applied penalized regression using data augmentation. ICV-adjusted WMH volume in adults, the number of deep and the number of periventricular WMH were dichotomized by median split. OR for CD4+ T-lymphocyte nadir is per 100 cells/μL. Abbreviations: CI = confidence interval; HIV = human immunodeficiency virus; ICV = intracranial volume; OR = odds ratio; VL = viral load (logarithmic value; unit: copies/ml) WMH = white matter hyperintensities

## Discussion

This study compared characteristics of cerebral WMH between children and adults living with HIV. Compared to children, adults showed a higher prevalence of WMH and had more WMH, regarding volume and number. This study further demonstrated that adults had both periventricular and deep WMH, whereas children had predominantly deep WMH.

The prevalence of WMH in children observed herein is similar to what was reported in another study, despite their inclusion of younger children who were suspected of having HIV-associated neurological complications, and were scanned with a less sensitive MRI [16]. Apart from our own earlier report [3], a recent study performed in Zambia found a comparable prevalence of WMH (18% vs. 24%) between PHIV children and HIV-negative controls, respectively [15]. The difference in prevalence might be explained by different study population and lower strength of magnetic field. Besides the prevalence, this study does not provide further specification of WMH, such as volume. Uncontrolled studies of HIV-negative children with other morbidities such as migraine, have reported a lower prevalence of WMH (up to 17%) [31, 32]. We found no association between WMH and age at treatment initiation in children, which is of interest as white matter maturation is at a crucial stage in early life [33]. We have incomplete data of possible disruption of treatment in the first two years of life, due to the majority of children arriving in the Netherlands at older age.

The median WMH volume observed in our adult cohort is similar to what has been reported in another study investigating a comparable group of adults living with HIV [17].

A notable finding from our analysis was the increased prevalence and number of periventricular, but not deep, WMH in adults compared to children. As both groups have a similar duration of known HIV infection, the observed prevalence and number of periventricular WMH in adults is likely at least partially explained by the higher age and higher prevalence of hypertension amongst our adult cohort participants [20]. The number of periventricular WMH in adults should be interpreted with caution, as some of these lesions tend to coalesce and are larger than ten millimeters, i.e. the commonly used border to distinguish between periventricular and deep lesions. In contrast to adults [4, 34], we failed to identify studies using visual rating scales (VRS), such as the Fazekas scale, to assess WMH in children. This is the reason for not using this scale in our study.

Children have predominantly deep WMH and due to this apparent difference with adults, we performed additional analyses in children to further explore potential associations not investigated in our previous study [3]. It could be hypothesized that cross-sectional associations might exist between certain risk factors (i.e. CDC classification or adoption status) or even clinical outcomes (total IQ), and prevalence of deep WMH; however, we found no such association.

The association with CD4^+^ T-lymphocyte nadir and periventricular WMH in adults suggests a possible role of HIV in the pathogenesis of these lesions. Previously published studies comparing adults with age-matched controls have generally found that HIV-positive status is independently associated with a higher WMH volume [4, 5, 17]. Two smaller studies however did not report similar findings [18, 35]. Even though the HIV-associated pathophysiology of WMH is incompletely understood, evidence suggests that WMH in HIV-negative participants are a consequence of small vessel disease [36]. Periventricular WMH are associated with older age and higher systolic blood pressure [37]. The etiology of WMH that are specifically localized deeply is not yet unraveled, though differences in clinical outcomes justify its distinction from periventricular WMH [38].

In our analysis, we found male gender to be a positively associated with the presence of deep WMH in children. The few studies investigating WMH in children have not investigated gender-associated WMH differences [3, 16]. This finding might suggest a potential protective role of estrogen on brain development. This hypothesis is supported by another study which found higher rates of progression and volume of WMH in older (postmenopausal) HIV-negative women as compared to men [39]. However, our finding could also be the artifact of inflated type 1 error from sparse data, yet we did apply penalized regression techniques to reduce this bias. Nevertheless, larger studies would be needed to further investigate the potential association between gender and WMH in HIV-infected individuals.

### Strengths and limitations

A strength of our analysis is that advanced non-invasive MRI imaging in both groups was performed using the same technique. We re-assessed all the segmentation to further harmonize the methodology. Our finding that the presence and location of WMH differs between children and adults living with HIV provides further insight into the distribution of WMH in these groups, hinting toward a different pathogenesis. Our study also has a number of important limitations. Although the independent association between positive HIV-status and higher WMH volume has been reported previously, the question of whether the difference in WMH distribution observed between children and adults, is associated with HIV remains unanswered, as we did not include HIV-negative participants. However, we aimed to exploratively assess the distribution of WMH in HIV-positive participants and hence we explicitly did not include a group of HIV-negative participants. As common to all cross-sectional studies, we were unable to establish whether the identified factors are causally linked to WMH [40]. Although we have recorded the medical history and for children the CDC stages as much as possible, medical historical information (e.g. prematurity or infections at early age) lacks from adults, and from children who arrived in The Netherlands at older age. Furthermore, the small number of pediatric participants reduces the power to detect risk factors. Lastly, the adult group contains only males and number of children was rather limited, both of which could affect the generalizability of our findings.

## Conclusions

In our cross-sectional assessment, we observed that adults have a higher prevalence, volume and number of total WMH compared to children living with HIV. This study suggests that the location of WMH differs between children and adults, suggesting a potentially different underlying pathogenesis. Future investigation including female adults could further explore potential gender differences in WMH prevalence. Nowadays, effectively treated children living with HIV in high resource settings are expected to live far into adulthood [41]. Therefore, it would be of interest to continue follow-up of these individuals during their life course and compare them to matched HIV-negative controls as well as to others who behaviorally acquired HIV. This information could help shed light on the evolution of WMH and its possible clinical effects.

## Supporting information

S1 Table (DOCX)

S2 Table WMH in pediatric participants (exclusion of three participants with a history of CNS infection). (DOCX)

S3 Table Univariable logistic regression analyses on ICV-adjusted WMH volume and location in children (exclusion of three participants with a history of CNS infection). (DOCX)
