# Supplementary material for: Differences in location of cerebral white matter hyperintensities in children and adults living with a treated HIV infection: A retrospective cohort comparison
Source: PLoS One. 2020 Oct 28;15(10):e0241438. doi: 10.1371/journal.pone.0241438 (PMC7592958; doi:10.1371/journal.pone.0241438)
Supplement: S1 Table — (DOCX) [file pone.0241438.s001.docx]

| **S1 Table.** | |
| --- | --- |
|  | **Children (n = 31)** |
| **Characteristics** |  |
| Adopted | 3 (10) |
| Total IQ | 75 (67.5 - 83) |
| **CDC classification** |  |
| CDC NA | 9 (29) |
| CDC B | 13 (42) |
| CDC C | 9 (29) |
| Variables used for analyses shown in table 3. Values noted in amount and percentage n(%) or median and inter-quartile range (IQR) Abbreviations: IQ = Intelligence Quotient, CDC = Center of Disease Control and prevention, NA = no to minimal symptoms; B moderate symptoms; C = severe symptoms or AIDS | |
