# Supplementary material for: Differences in location of cerebral white matter hyperintensities in children and adults living with a treated HIV infection: A retrospective cohort comparison
Source: PLoS One. 2020 Oct 28;15(10):e0241438. doi: 10.1371/journal.pone.0241438 (PMC7592958; doi:10.1371/journal.pone.0241438)
Supplement: S2 Table — (DOCX) [file pone.0241438.s002.docx]

**S2 Table. WMH in pediatric participants (exclusion of three participants with a history of CNS infection).**

|  | **Participants without CNS infection (n=24)** | **Adults (n=74)** | ***p* value** |
| --- | --- | --- | --- |
| Total volume (IQR) | 106 mm^3^ (60.8 – 524) | 1182 mm^3^ (425 – 2617) | <0.001 |
| Number of deep WMH (IQR) | 3 (2 – 11.5) | 11 (3.0 – 27.8) | 0.12 |
| Number of periventricular WMH (IQR) | 1 (0 – 2.0) | 7.0 (6.0 – 10.8) | <0.001 |

Abbreviations: CNS = central nervous system; IQR = interquartile range; WMH = white matter hyperintensities
