# Supplementary material for: Differences in location of cerebral white matter hyperintensities in children and adults living with a treated HIV infection: A retrospective cohort comparison
Source: PLoS One. 2020 Oct 28;15(10):e0241438. doi: 10.1371/journal.pone.0241438 (PMC7592958; doi:10.1371/journal.pone.0241438)
Supplement: S3 Table — (DOCX) [file pone.0241438.s003.docx]

| **S3 Table. Univariable logistic regression analyses on ICV-adjusted WMH volume and location in children (exclusion of three participants with a history of CNS infection)** | | | | | | |
| --- | --- | --- | --- | --- | --- | --- |
|  | ICV-adjusted WMH volume in children (higher versus lower than median) | | Presence of deep WMH in children (yes versus no) | | Presence of periventricular WMH in children (yes versus no) | |
|  | **OR (95% CI)** |  | **OR (95% CI)** |  | **OR (95% CI)** |  |
| Age | 1.00 (0.75 - 1.32) |  | 0.88 (0.66 - 1.16) |  | 0.96 (0.73 - 1.28) |  |
| Female gender | 0.36 (0.08 - 1.61) |  | 0.17 (0.03 - 0.81) |  | 0.81 (0.19 - 3.46) |  |
| High blood pressure | 1.39 (0.22 - 8.91) |  | 3.59 (0.38 - 33.73) |  | 2.70 (0.40 - 18.47) |  |
| Known HIV years | 1.33 (0.84 - 2.10) |  | 1.17 (0.78 - 1.74) |  | 1.59 (1.01 - 2.50) |  |
| Age at treatment initiation | 1.10 (0.88 - 1.39) |  | 1.02 (0.83 - 1.26) |  | 1.11 (0.90 - 1.37) |  |
| Treatment years | 0.90 (0.74 - 1.08) |  | 0.94 (0.79 - 1.11) |  | 0.90 (0.75 - 1.07) |  |
| CD4^+^ nadir *Z* score | 0.61 (0.25 - 1.46) |  | 0.70 (0.32 - 1.56) |  | 0.44 (0.14 - 1.39) |  |
| HIV VL zenith | 0.47 (0.11 - 2.10) |  | 0.85 (0.22 - 3.31) |  | 0.90 (0.23 - 3.60) |  |
|  |  |  |  |  |  |  |
| CDC NA |  |  | 1.66 (0.28 - 10.05) |  | 0.29 (0.03 - 2.67) |  |
| CDC B |  |  | 0.47 (0.47 - 2.24) |  | 1.41 (0.29 - 6.86) |  |
| CDC C |  |  | 166. (0.28 - 10.05) |  | 1.80 (0.31 - 10.52) |  |
| Adopted |  |  | 0.85 (0.11 - 6.33) |  | 1.53 (0.20 - 11.84) |  |
| Total IQ |  |  | 1.05 (0.98 - 1.12) |  | 1.05 (0.98 - 1.13) |  |
| Univariable logistic regression models with applied penalized regression using data augmentation. ICV-adjusted WMH volume in children was dichotomized by median split. High blood pressure measured at enrollment defined per guidelines of American Association of Pediatrics; CD4^+^ nadir *Z* score is age-adjusted. Abbreviations: CDC = Center for Disease Control and Prevention, A = minimal symptoms to AIDS, B = moderate symptoms C = severe symptoms or AIDS; HIV = human immunodeficiency virus; ICV = intracranial volume; IQ = intelligent quotient; OR = odds ratio; VL = viral load (logarithmic value; unit: copies/ml); WMH = white matter hyperintensities | | | | | | |
|  |  |  |  |  |  |  |
|  |  |  |  |  |  |  |
|  |  |  |  |  |  |  |
